# Dengue-1 Virus Isolation during First Dengue Fever Outbreak on Easter Island, Chile

**DOI:** 10.3201/eid0911.020788

**Published:** 2003-11

**Authors:** Cecilia Perret, Katia Abarca, Jimena Ovalle, Pablo Ferrer, Paula Godoy, Andrea Olea, Ximena Aguilera, Marcela Ferrés

**Affiliations:** * Pontificia Universidad Catolica de Chile School of Medicine, Virology Laboratory, Santiago, Chile; † Ministry of Health, Santiago, Chile

## Abstract

Dengue virus was detected for the first time in Chile, in an outbreak of dengue fever on Easter Island. The virus was isolated in tissue culture and characterized by reverse transcription–polymerase chain reaction as being dengue type 1.

Dengue fever (DF) is a common viral disease of the tropics. Only a few countries in the Americas, including Chile, have not reported cases of this disease. Distribution of dengue virus in the Americas has increased since 1970, when efforts to eradicate the vector (*Aedes aegypti*) waned, particularly in Central America and the Amazon region. Not only has the number of DF cases on the continent (*1*) increased but all four types of dengue virus have also been introduced. Consequently, the number of dengue hemorrhagic fever cases (DHF) has risen because secondary infections are now common in populations in which multiple dengue serotypes are circulating.

At the beginning of 20th century, *A. aegypti* existed in northern Chile, where the climate is suitable for the mosquito to breed, but it was eradicated in 1945 (*2*). Since then, no evidence of reintroduction of the mosquito was observed by entomologic surveillance. However, by the end of 2000, the presence of the mosquito was confirmed on Easter Island (*3*), which is located in the Pacific Ocean 3,800 km off the coast of Chile. All of the island’s 3,860 inhabitants live in one village, Hanga Roa, on the western coast. At that time, 70% of the houses of this village were infested by *A. aegypti,* according to studies performed by the Epidemiological Unit of the Ministry of Health (*4*). Devices to catch mosquito larvae were installed in a sampling of houses, in the rural sectors, and near the three volcano lakes. The larvae were found in the entire urban sector, in some sections of the rural areas (Vaitea y Tahai), and in none of the volcano lakes (*5*). Educational campaigns and control efforts (insecticides and reduction of container breeding sites) were carried out to decrease mosquito infestation. During the 2002 dengue outbreak, an average of 5% of the sampled houses were infested.

Before the outbreak on Easter Island, 15 cases of DF had been diagnosed in continental Chile in 2000 and 2001 and serologically confirmed in our laboratory. Dengue was acquired for all case-patients during when traveling within the American continent.

## The Study

The index case-patient, a 21-year-old Chilean woman, had been living on Easter Island for 2 months and had not traveled. She had a high temperature (39°C), myalgias, arthralgias, headache, and a maculopapular rash for 7 days. Laboratory analysis of a blood sample indicated low leukocyte and platelet counts. While still febrile, she traveled to Santiago, the capital of Chile, and was admitted to a private hospital; DF was suspected. On March 13, 2002, DF was confirmed by an in-house dengue immunoglobulin (Ig) M enzyme-linked immunosorbent assay (ELISA) in our laboratory. This case of DF was the first acquired in Chile.

The Ministry of Health organized an outbreak investigation team. As part of the study and with the goal of recovering and identifying the virus, blood samples were taken from 16 febrile patients who were assessed and satisfied the clinical definition of suspected dengue case made by the Ministry of Health. The samples were brought to our laboratory, and plasma was used for viral culture and for reverse transcription–polymerase chain reaction (RT-PCR). Serologic testing was not performed on these samples.

Viral culture was attempted from 15 acute-phase plasmas. Plastic flasks (T-25) seeded with Vero cells were injected with 200 μL of plasma diluted 1:5 with medium 199, 2% fetal bovine serum, gentamycin 50 μg/mL. After 1 hour of absorption at 37°C, cultures were incubated 10 days at the same temperature and observed once a day for cytopathic effect (CPE). Cells were harvested for indirect immunofluorescence antibody testing (IFAT) after CPE was first observed (as early as day 5 in some of the cultures and on day 10 of incubation in all the other samples). Initially, IFAT was performed with polyclonal antisera reactive with all serotypes (D1–D4); then samples with positive results were stained with monoclonal antibodies specific for each subtype to identify dengue serotypes.

A nested RT-PCR developed by Lanciotti (*6*) was used to analyze plasma and viral culture supernatants from 15 febrile patients. Samples (200 μL) were taken, and RNA was extracted with Trizol (Gibco BRL, Life Technologies, Rockville, MD). RNA (5 μL) was reverse transcripted and cDNA amplified with primers D1 and D2, SuperScript II, *Taq* polymerase (Gibco) in a single reaction vessel with 50 μL final volume. The thermocycler was programmed to incubate for 1 h at 42°C and then 35 cycles at 94°C, 55°C, and 72°C. The second step used 10 μL of diluted 1:100 dengue cDNA from the first reaction and contained primers to amplify the four dengue serotypes (TS1–TS4, plus D1). The results had bands of different sizes, depending on the serotype (DENV-1 482 bp, DENV-2 119 bp, DENV-3 290 bp and DENV-4 392 bp) after 20 cycles at the same temperatures as the first reaction.

**Figure 1:**
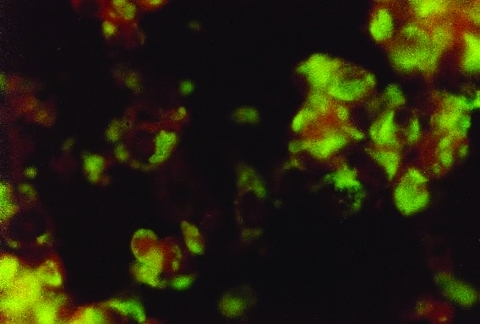
Indirect immunofluorescence antibody testing with monoclonal antibodies identifying dengue-1 virus in tissue culture of Vero cells.

**Figure 2:**
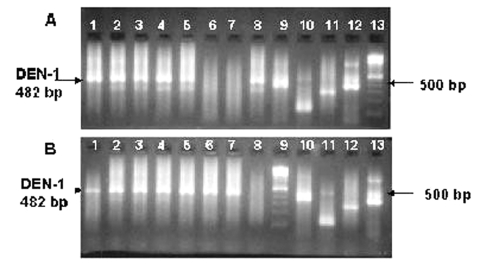
Visualization of reverse transcription–nested polymerase chain reaction product from 15 cultures of supernatant. DENV-1 positive samples are indicated by a 482-bp band. A) Lanes 1–5: positive culture supernatants. Lanes 6–7: negative culture supernatants. Lane 8: positive culture supernatant. Lane 9: positive DENV-1 control. Lane 10: positive DENV-2 control. Lane 11: positive DENV-3 control. Lane 12: positive DENV-4 control. Lane 13: 100-bp DNA ladder. B) Lanes 1–7: positive culture supernatants. Lane 8: negative control. Lane 9: 100-bp DNA ladder. Lane 10: positive DENV-1 control. Lane 11: positive DENV-2 control. Lane 12: positive DENV-3 control. Lane 13: positive DENV-4 control.

Dengue virus was isolated from 13 of 15 acute-phase plasmas by viral culture. One of the negative plasma samples was from a patient who was febrile for 5 days. The isolated dengue virus was identified as DENV-1 serotype by IFAT by using monoclonal antibodies in slides prepared from the viral cultures (Figure 1) and by RT-PCR obtaining a band of 482 bp (Figure 2).

When Lanciotti primers design was used, RT-PCR amplified virus RNA from the 13 positive cell culture supernatants but from none of the acute-phase plasmas. To improve sensitivity, the primer TS1 was modified, decreasing the Cs and Gs at the 3′ end. The new primer was located in the genome position 575–595, instead of 568–586 (*7*), amplifying a DNA product of 491 bp. Using this new TS1 primer, we could amplify dengue-1 RNA in 8 of 15 plasmas; none of the negative cultures plasmas was positive by PCR.

In addition to the virologic study, a serum sample was taken from 423 asymptomatic convalescent patients who recalled being febrile during the last 2 months. These samples were tested for dengue IgM by ELISA at the National Reference Laboratory of the Ministry of Health; 176 were IgM positive.

According to the epidemiologic results, the outbreak was from January to May 2002, and 636 cases of DF were diagnosed. A total of 460 cases were diagnosed by epidemiologic nexus, satisfying the case definition, and 176 were confirmed by IgM serologic testing. Therefore, the incidence rate of the disease was 16.6% (*4*). No cases of DHF were diagnosed.

## Conclusions

The isolation of the virus from febrile patients during the outbreak confirmed the first appearance of dengue virus in insular Chile and the fact that the virus causing the epidemic is DENV-1. The identification of the virus has allowed us to presume that the original source of the virus might be tourists from either Brazil or Tahiti. Most of the tourists (45%) visiting Easter Island came from Brazil. A lower proportion came from the Pacific Islands, where the same virus serotype was circulating at the time the outbreak started. Knowing the serotype is important to keep a strict surveillance of febrile patients and mosquitoes to determine if a different dengue virus serotype is introduced and to determine if cases of DHF are appearing on the island.

Laboratory tests, like serology (IgM and IgG ELISA), and RT-PCR for dengue virus, were already available at our laboratory, whereas viral culture with IFAT for virus identification was quickly developed when the DF outbreak was identified. The further genotyping of the isolated dengue virus will allow us to compare with other DENV-1 viruses circulating in other parts of the world and determine the origin of Easter Island DENV-1.

Because of diagnosis of the first indigenous case of DF in a country where tropical infections are unusual, being able to make a differential diagnosis and having laboratory resources for a variety of emerging infectious diseases are important, particularly for an immunologically naive community, such was the case of Easter Island and the Chilean population.
